# Soil diazotrophic abundance, diversity, and community assembly mechanisms significantly differ between glacier riparian wetlands and their adjacent alpine meadows

**DOI:** 10.3389/fmicb.2022.1063027

**Published:** 2022-12-08

**Authors:** Danhong Chen, Haiyan Hou, Shutong Zhou, Song Zhang, Dong Liu, Zhe Pang, Jinming Hu, Kai Xue, Jianqing Du, Xiaoyong Cui, Yanfen Wang, Rongxiao Che

**Affiliations:** ^1^Yunnan Key Laboratory of International Rivers and Transboundary Eco-Security, Institute of International Rivers and Eco-Security, Yunnan University, Kunming, China; ^2^School of Ecology and Environmental Science, Yunnan University, Kunming, China; ^3^College of Life Sciences, University of Chinese Academy of Sciences, Beijing, China; ^4^State Key Laboratory of Pollution Control and Resource Reuse, School of the Environment, Nanjing University, Nanjing, China; ^5^Yunnan Key Laboratory for Plateau Mountain Ecology and Restoration of Degraded Environments, School of Life Sciences, Yunnan University, Kunming, China

**Keywords:** nitrogen fixers, riparian wetlands, alpine meadows, Qinghai–Tibet Plateau, global warming

## Abstract

Global warming can trigger dramatic glacier area shrinkage and change the flux of glacial runoff, leading to the expansion and subsequent retreat of riparian wetlands. This elicits the interconversion of riparian wetlands and their adjacent ecosystems (e.g., alpine meadows), probably significantly impacting ecosystem nitrogen input by changing soil diazotrophic communities. However, the soil diazotrophic community differences between glacial riparian wetlands and their adjacent ecosystems remain largely unexplored. Here, soils were collected from riparian wetlands and their adjacent alpine meadows at six locations from glacier foreland to lake mouth along a typical Tibetan glacial river in the Namtso watershed. The abundance and diversity of soil diazotrophs were determined by real-time PCR and amplicon sequencing based on *nifH* gene. The soil diazotrophic community assembly mechanisms were analyzed *via* iCAMP, a recently developed null model-based method. The results showed that compared with the riparian wetlands, the abundance and diversity of the diazotrophs in the alpine meadow soils significantly decreased. The soil diazotrophic community profiles also significantly differed between the riparian wetlands and alpine meadows. For example, compared with the alpine meadows, the relative abundance of chemoheterotrophic and sulfate-respiration diazotrophs was significantly higher in the riparian wetland soils. In contrast, the diazotrophs related to ureolysis, photoautotrophy, and denitrification were significantly enriched in the alpine meadow soils. The iCAMP analysis showed that the assembly of soil diazotrophic community was mainly controlled by drift and dispersal limitation. Compared with the riparian wetlands, the assembly of the alpine meadow soil diazotrophic community was more affected by dispersal limitation and homogeneous selection. These findings suggest that the conversion of riparian wetlands and alpine meadows can significantly alter soil diazotrophic community and probably the ecosystem nitrogen input mechanisms, highlighting the enormous effects of climate change on alpine ecosystems.

## Introduction

The unprecedented global warming causes glacier melting, which leads to the extension of glacial riparian wetlands. However, the melting of glaciers will finally decrease the glacier volume, eliciting the retreat of glacial riparian wetlands (Vargo et al., 2020; Wang et al., 2020b; Zhang et al., 2021b). Nitrogen is the predominant factor limiting the terrestrial primary productivity, and thus its input rates can largely determine the ecosystem carbon fixation ability and feedbacks to global warming (Mack et al., 2004; LeBauer and Treseder, 2008). Biological nitrogen fixation is the main nitrogen source in most natural ecosystems (Cleveland et al., 1999; Vitousek et al., 2013). As the main drivers of biological nitrogen fixation, soil diazotrophic abundance and diversity are critical controllers of soil nitrogen fixation activity (Reed et al., 2010; Zhang et al., 2020; Wu et al., 2021). Therefore, exploring the differences in soil diazotrophic abundance and diversity between glacial riparian wetlands and their adjacent ecosystems can provide critical insights to understanding alpine ecosystem nitrogen cycling feedbacks to global warming.

In recent decades, soil diazotrophs are extensively investigated, attributing to the population of amplicon high throughput sequencing based on *nifH* gene (Zehr et al., 2003; Zhang et al., 2017; Gaby et al., 2018; Knight et al., 2018). Accordingly, our understanding of soil diazotrophic distribution patterns and their responses to environmental changes and human activities has been largely improved (Che et al., 2018a; Kuypers et al., 2018; Chen et al., 2019; Fan et al., 2019; Tang et al., 2021). The primary productivity of meadows and wetlands is usually highly limited by nitrogen availability (Venterink et al., 2002; Stevens et al., 2015). Accordingly, biological nitrogen fixation and soil diazotrophs are also increasingly studied in these ecosystems (Larmola et al., 2014; Wang et al., 2017; Xiao et al., 2020). In particular, due to climate change and anthropogenic disturbance, large areas of wetlands are degraded to meadows, and thus the responses of soil diazotrophs to wetland degradation are extensively investigated (Wu et al., 2016; Li et al., 2022b; Wang et al., 2022a). For instance, the degradation of marshes to meadows could significantly decrease soil diazotrophic diversity and substantially change soil diazotrophic community profiles, which were mainly influenced by total organic carbon, moisture, and total nitrogen (Li et al., 2022a). The significant effects of peatland degradation on soil diazotrophic abundance, diversity, and activity were also observed (Zhang et al., 2020). As mentioned above, global warming can also lead to the extension of glacial riparian wetlands, converting the alpine meadows into wetlands (Cao et al., 2015; Ayub et al., 2020). However, most of the existing studies only concern the responses of soil diazotrophs to wetland degradation. There is still a paucity of knowledge on understanding the soil diazotrophic responses to the interconversion of meadows and wetlands.

The soil diazotrophs in glacial riparian wetlands and their adjacent alpine meadows probably substantially differ due to the following reasons. First, the contents of soil moisture and organic carbon in wetlands are usually significantly higher than those in their adjacent alpine meadows (Fisher and Acreman, 2004). Thus, based on the generally copiotrophic life strategy of diazotrophs, the abundance and diversity of diazotrophs in riparian wetlands would be significantly higher than those in their adjacent alpine meadows. Second, the differences in soil pH and redox potential between wetlands and meadows are also frequently observed (Zou et al., 2009; Xing et al., 2022). Third, the vegetation cover and plant community profiles in wetlands and meadows are usually substantially different (Patten, 1998; Gao et al., 2015; Jiang et al., 2021). As is widely recognized, soil moisture and nutrient contents, pH, redox potential, and plant community are key environmental factors shaping soil diazotrophic community (Tang et al., 2017; Wang et al., 2017, 2022b; Li et al., 2021a; Rui et al., 2022). Thus, the differences in the aforementioned environmental factors between wetlands and their adjacent meadows can dramatically affect soil diazotrophic communities (Zhu et al., 2022). In the alpine regions, highly abundant and diverse diazotrophs have been observed in wetland soils (Larmola et al., 2014; Che et al., 2018a; Terra et al., 2022). Furthermore, as mentioned above, the degradation of wetlands can significantly decrease soil diazotrophic diversity and activity (Li et al., 2022a; Wang et al., 2022a). Hence, there would be significant differences in the community composition and assembly mechanisms of diazotrophs between riparian wetlands and their adjacent alpine meadows.

This study aimed to reveal the differences in soil diazotrophic abundance, diversity, and community assembly mechanisms between glacial riparian wetlands and their adjacent alpine meadows. Six sampling sites were set along the Niyaqu, a typical Tibetan glacial river in the Namtso watershed. Soil samples were collected from the riparian wetland and its adjacent alpine meadow at each site. The abundance and community profiles of soil diazotrophs were determined by real-time PCR and amplicon high-throughput sequencing of the *nifH* gene. The community assembly mechanisms were quantified using iCAMP (Infer Community Assembly Mechanisms by Phylogenetic bin-based null model analysis), a recently developed null model algorithm based on phylogenic binning (Ning et al., 2020). Functional profiles were predicted based on FAPROTAX (Functional Annotation of Prokaryotic Taxa; Louca et al., 2016).

## Materials and methods

### Study sites and soil collection

The soils of six riparian wetlands and their adjacent alpine meadows were collected from glacier foreland to lake mouth along the 35-km riparian zone of the Niyaqu, a typical Tibetan glacial frontier river in the Namtso watershed, in mid-August 2019 (Supplementary Figure 1). The Namtso basin has a semiarid subarctic plateau climate and the elevation of the Niyaqu catchment is 4732 to 5703 m, covering an area of 388 km^2^ (Yu et al., 2019). This study area is featured by high altitude, cold climate, and large diurnal temperature difference (Zhou et al., 2022). The mean annual precipitation and mean annul temperatures are 35 mm and 2.8°C, respectively. Based on Soil Units in the Revised Legend of the Soil Map of the World (FAO90), at the first two sites, the soil type is Gelic Leptosols, while it is Haplic Phaeozems at the last four sites. The plant communities in the riparian wetlands were dominated by *Kobresia littoralis*, *Kobresia tibetana*, and *Kobresia humilis*, while those in the alpine meadows were dominated by *Kobresia*, *Carex*, and *Potentilla bifida*.

The riparian soils were collected from the wetlands near the Niyaqu, while the meadow soils were collected from the alpine meadows with perpendicular distance of approximately 200–500 m from the wetlands. At least in the short term, the alpine meadows are unaffected by the glacial runoff. We set three and five sampling replications for Site 1 and the other sites, respectively. At each site, the sampling replications were set in a straight line with the distances between the adjacent plots being more than 10 m. As for each sampling replication, five soil subsamples of 0–20 cm depth were collected using a standard soil corer (7 cm in diameter). Then, the soil subsamples were sieved to ≤2 mm and composited into one sampling replication. In total, we obtained 56 composite soil samples. Then, the soil samples were divided into three subsamples which were preserved at −80°C, 4°C, and room temperature (air-dried) for different usage purposes, respectively. Additionally, we used GPS to record the longitude and latitude of the sampling points. The plant coverage and the sub-coverage of each plant species in each quadrat were estimated by an expert. The species richness was obtained by calculating the total number of plant species. Then, the aboveground plant biomass in a 0.5 m × 0.5 m plot was collected using garden shears.

### The analysis of soil and plant properties

The soils were dried at 105°C for 48 h to determine moisture contents. With a soil to water mass ratio of 1:2.5, the pH value of the soil was measured with a pH meter (Shi et al., 2021). The contents of soil total carbon, total organic carbon, and total nitrogen were measured by the auto elemental analyzer (Che et al., 2017). The chloroform fumigation extraction method was used to measure the contents of microbial biomass carbon and nitrogen (Brookes et al., 1985). The contents of soil total phosphorus and potassium were determined by alkali fusion-Mo-Sb Anti spectrophotometric (HJ 632-2011) and flame photometric methods (Pratt, 1965), respectively. The contents of ammonium nitrogen and nitrate nitrogen in the soils were determined by indophenol blue and vanadium chloride chromogenic method (Lu, 2000; Huang and Zhan, 2002). According to the soil pH value, the available phosphorus content of the soils was determined by Bray I method (Bray and Kurtz, 1945). The plant aboveground biomass was measured by drying at 65°C for 48 h (Sairam et al., 2002). The basic properties of the soils and plants are shown in Supplementary Table 1.

### The DNA extraction and NovaSeq sequencing

The soil DNA was extracted using the DNeasy PowerSoil^®^ Kit (Qiagen, Hilden, Germany), following the manufacturer’s instructions. The *nifH* gene was amplified using PCR with barcoded universal primer sets (PolF: TGC GAY CCS AAR GCB GAC TC; PolR: ATS GCC ATC ATY TCR CCG GA; Poly et al., 2001). The PCR reaction mixture (50 μL) contained: 1 μL of template DNA, 25 μL of Premix Taq Hot Start Version (Takara Bio Inc., Shiga, Japan), 1 μL of each primer (20 μM), and 22 μL of nuclease-free water. The PCR runs started with an initial denaturation and enzyme activation step at 95°C for 10 min, followed by 40 cycles of 95°C for 30 s, 56°C for 30 s, and 72°C for 45 s, and finally extended at 72°C for 10 min. The GeneJET Gel Extraction Kit (Thermo Scientific, Lithuania) was used to purify the PCR products, and then we pooled all the PCR products with equal molality for each sample (Che et al., 2018a). Finally, the Illumina NovaSeq high-throughput sequencing was performed by Magigene Co., Ltd (Guangdong, China) with a paired-end strategy and the sequencing depth was over 30000 sequences per sample (Supplementary Figure 4).

### Bioinformatics analysis

The *nifH* sequence data were mainly processed with tools including USEARCH (v11; Edgar, 2013), Mothur (v1.27; Schloss et al., 2009), and BLAST (v2.13.0). Specifically, the raw paired-end sequences of all the samples were merged using USEARCH into a fastq file. Then, the search_pcr2 script was used to remove the forward and reverse primers, and the maxdiffs parameter was set to 2 to delete sequences with primer mismatches of more than 2 (Edgar, 2013). Based on the fastq_filter script with the fastq_truncqual parameter of 20, the sequences with quality scores less than 20 were removed. Subsequently, we used the fastx_uniques script to generate unique sequence dataset. ZOTU representative sequences were generated using the UNOISE3 non-clustering denoising algorithm, and ZOTUs with sequence numbers less than 9 were removed (Edgar, 2016). The chimeras of ZOTU representative sequences were removed using the uchime2_ref command. The chimera-free ZOTU representative sequences were aligned to the nitrogen-fixing gene sequence database (Gaby and Buckley, 2014) in Mothur, and the unaligned sequences were removed (Schloss et al., 2009). Then, the clean representative sequences were clustered with 91.9% similarity threshold using the cluster_smallmem command in Usearch and OTU representative sequences were generated (Gaby et al., 2018). Finally, the otutab script was used to generate the OTU table. The taxonomic annotation of *nifH* genes were performed *via* the online blastx tool with the NCBI database (*E*-value ≤ 10^–5^, identity ≥ 90%), and only the successfully annotated OTUs were included in the following analysis. The samples without enough sequencing depth have been removed from the analysis. In total, 313 OTUs were obtained for the 53 soil samples, and the sequence number of each sample was rarefied to 17711 for all the subsequent analysis. The diazotrophic richness was calculated in R with the vegan package. Additionally, the functional profiles of the diazotrophs were predicted based on FAPROTAX. All the raw sequences were deposited to the NCBI Sequence Read Archive under the BioProject PRJNA417160, with accession number from SAMN31867209 to SAMN31867264.

### The determination of diazotrophic community assembly mechanism

We analyzed the relative effects of different ecological processes using the iCAMP (Ning et al., 2020) package in R (R Development Core Team, 2022). This method exhibits high accuracy (0.93–0.99), precision (0.80–0.94), sensitivity (0.82–0.94), and specificity (0.95–0.98) in determining microbial community assembly mechanisms (Stegen et al., 2013; Ning et al., 2020). The phylogenetic bins were constructed based on the phylogenetic tree which was generated through the “phylogeny” script in QIIME2 (Bolyen et al., 2019). The phylogenetic tree was truncated at a certain phylogenetic distance from the root (as short as possible) at which the connections between all species were below a threshold *d*_*s*_ (0.2). Taxa derived from the same ancestor after the cutoff point were grouped into the same strict bin. Then each small bin (*n*_*taxa*_ < 24) was merged into its nearest bin. This process was repeated until all the merged bins were of sufficient size (*n*_*min*_ ≥ 24). To evaluate the phylogenetic signal of each bin, we used Mantel test to evaluate the correlations between phylogenetic distance and niche differences of each species in a bin. The bins with correlations of *r* > 0.1 and *P* < 0.05 were defined to have significant phylogenetic signals. Then, the effects of different ecological processes on the subcommunity assembly of each bin were calculated. Finally, in each bin, according to the pairwise βNRI and RC, the ecological process dominating the turnovers between each pair of soils was determined. The turnovers of diazotrophic community pairs with βNRI > 1.96 are dominated by heterogeneous selection; the turnovers of diazotrophic community pairs with βNRI < −1.96 are mainly controlled by homogeneous selection; the turnovers of diazotrophic community pairs with | βNRI| ≤ 1.96 and RC > 0.95 are mainly controlled by the dispersal limitation; the turnovers of diazotrophic community pairs with | βNRI| ≤ 1.96 and RC < −0.95 are mainly controlled by homogenizing dispersal; and the turnovers of diazotrophic community pairs with | βNRI| ≤ 1.96 and | RC| ≤ 0.95 are mainly controlled by drift (Ning et al., 2020).

### Statistical analysis

Based on the two-way nested ANOVA and Duncan’s *post-hoc* test, the differences in soil properties, plant properties, diazotrophic abundance, and diazotrophic diversity between riparian wetlands and alpine meadows, as well as among the study sites were analyzed. The non-metric multidimensional scaling (NMDS) based on Bray-Curtis distance was performed to visualize the differences in diazotrophic community profiles, which were further tested by permutational multivariate analysis of variance (PERMANOVA). The relationships between diazotroph community structure and environmental factors were examined based on the envfit function in vegan package. The Wilcox_test was used to analyze the differences in the relative abundance of diazotrophic lineages between riparian wetland and alpine meadow soils at the taxonomic levels from kingdom to family. Then, the differences were visualized using GraPhlAn, and only the taxa with statistically significant differences were shown (Graphical Phylogenetic Analysis; v1.1.3; Asnicar et al., 2015). The relationships between the relative abundance of soil diazotrophic functional groups and soil properties were determined by the Pearson correlation analysis. Most of the statistical methods used in this study are robust to the inconsistent sample sizes. All the statistical analyses were performed using R (R Development Core Team, 2022).

## Results

### The soil and plant properties

As shown in Supplementary Table 1 and Supplementary Figure 2, the soil and plant properties showed different spatial trends for the riparian wetlands and alpine meadows. Particularly, in the riparian wetlands, many variables such as the contents of soil total carbon, total organic carbon, total inorganic carbon, total nitrogen, microbial biomass carbon, microbial biomass nitrogen, and moisture showed a first increase but then decrease trend from Site 1 to Site 6. Nevertheless, in the alpine meadows, these variables and plant coverage gradually decreased from Site 1 to Site 6. Additionally, most soil and plant properties significantly differed between the riparian wetlands and their adjacent alpine meadows. Specifically, the moisture contents were significantly higher in the riparian wetland soils except at Site 1. At Site 1, compared with the riparian wetlands, the alpine meadows exhibited significantly higher contents of soil total carbon, total inorganic carbon, and total nitrogen, as well as plant richness, but they had significantly lower soil available phosphorus content. At Site 2, soil total phosphorus and available phosphorus contents were significantly enriched in the alpine meadow soils. At Site 3, soil total potassium and available phosphorus contents were also more abundant in the alpine meadow soils. In addition, at Site 4, the contents of soil total carbon, total inorganic carbon, total nitrogen, microbial biomass nitrogen, and plant coverage were significantly higher in the riparian wetland soils than those in the alpine meadows. At Site 5, the contents of soil total carbon, total organic carbon, total inorganic carbon, total nitrogen, microbial biomass carbon, microbial biomass nitrogen, nitrate nitrogen, ammonium nitrogen, pH, and plant coverage were also significantly higher in the riparian wetlands, while soil total potassium showed an adverse trend. At Site 6, the contents of soil total carbon, total organic carbon, total nitrogen, microbial biomass carbon, microbial biomass nitrogen, plant coverage, and plant richness were also significantly higher in the riparian wetland soils, while soil ammonium nitrogen content and pH displayed an opposite trend.

### The soil diazotrophic abundance and diversity

**FIGURE 1:**
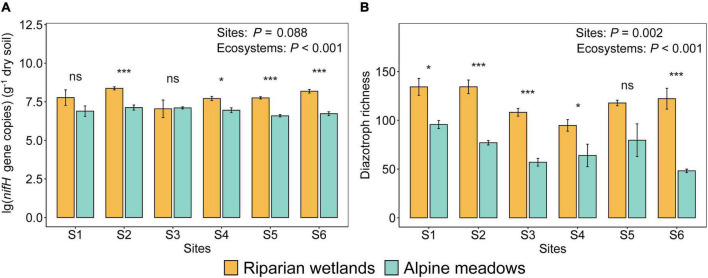
The differences in the diazotrophic abundance **(A)** and richness **(B)** between the riparian wetland and alpine meadow soils. All the data were presented as mean ± SE [Sites: The effects of sites; ecosystems: The effects of ecosystem types (i.e., alpine meadows and riparian wetlands); Asterisks indicate the significance of the ANOVA results (*P < 0.05; ***P < 0.001; ns: no significance)].

The abundance and richness of soil diazotrophs in the riparian wetlands were significantly higher than those in the alpine meadows (Figure 1; *P* < 0.001). In detail, the average *nifH* gene copies in the riparian wetland and alpine meadow soils were 6.05 × 10^7^−2.64 × 10^8^ and 4.14 × 10^6^−1.79 × 10^7^ per gram of dry soil, respectively (Figure 1A). The diazotrophic richness in the riparian wetland and alpine meadow soils were 95–134 and 48–96, respectively (Figure 1B). The diazotrophic abundance difference among the sites was not significant (*F* = 2.078; *P* = 0.088). In contrast, the diazotrophic richness varied significantly among the sites (*F* = 4.563; *P* = 0.002), with a trend of first decreasing and then increasing from the upstream to the downstream.

**FIGURE 2:**
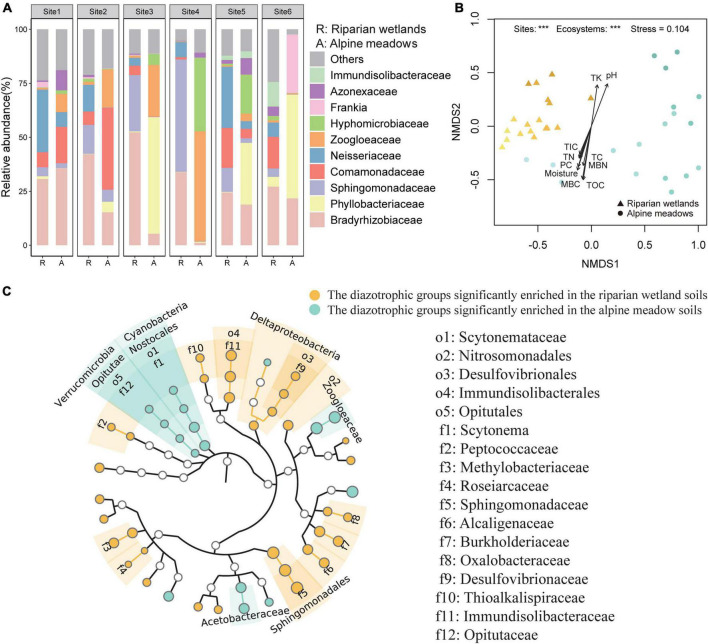
The differences in the soil diazotrophic community composition between the riparian wetlands and the alpine meadows. **(A)** Soil diazotrophic community compositions at the family level in the riparian wetlands and the alpine meadows. **(B)** The NMDS ordination combined with envfit analysis of diazotrophic community structure in the riparian wetland and the alpine meadow soils. TC, soil total carbon content; TOC, soil total organic carbon content; TIC, soil total inorganic carbon content; TN, soil total nitrogen content; TK, soil total potassium content; MBC, soil microbial biomass carbon content; MBN, soil microbial biomass nitrogen content, and moisture: soil moisture content; PC, plant coverage. **(C)** The differences in the relative abundance of diazotrophic taxa between the riparian wetland and alpine meadow soils at the taxonomic levels from phylum to genus. The differences were revealed through the Wilcoxon test (*P* < 0.05). ***P < 0.001.

Proteobacteria dominated the soil diazotrophic communities, accounting for more than 90% of the diazotrophic communities in both the riparian wetland and alpine meadow soils. The NMDS and PERMANOVA showed that the soil diazotrophic community composition was significant different between the riparian wetlands and alpine meadows (Figure 2B; *P* < 0.001). At the phylum and class levels, Cyanobacteria, Verrucomicrobia, Nostocales, and Opitutae were more abundant in the alpine meadow soils, while Deltaproteobacteria showed an opposite trend (Figure 2C). At the order level, the relative abundances of Sphingomonadales, Nitrosomonadales, Desulfovibrionales, and Immundisolibacterales were significantly higher in the riparian wetlands compared with those in the alpine meadow soils. In contrast, the proportion of Scytonemataceae showed an adverse trend. At the family level, Bradyrhizobiaceae (24.44–52.30%), Sphingomonadaceae (3.88–52.08%), and Neisseriaceae (3.62–28.99%) were the main diazotrophic taxa in the riparian wetland soils (Figure 2A). As for the alpine meadow soils, Phyllobacteriaceae (0.07–54.00%), Zoogloeaceae (0.30–50.77%), Bradyrhizobiaceae (1.20–35.70%), Comamonadaceae (0.13–37.93%), and Hyphomicrobiaceae (0.11–34.03%) dominated the diazotrophic communities. Further analysis suggested that the relative abundances of Methylobacteriaceae, Alcaligenaceae, Burkholderiaceae, Oxalobacteraceae, and unclassified_Frankia were significantly higher in the riparian wetland soils than those in the alpine meadow soils, while Acetobacteraceae and Zoogloeaceae showed reverse trends (Figures 2A,C). The community profiles were also significantly different among the study sites (Figure 2A). Specifically, Comamonadaceae was highly enriched in the alpine meadows at Site 2. At Site 3, the most abundant diazotrophic families were Bradyrhizobiaceae and Phyllobacteriaceae in the riparian wetland and alpine meadow soils, respectively. At Site 4, the riparian wetland diazotrophic community was dominated by Azonexaceae, whereas the alpine meadow diazotrophic community was dominated by Zoogloeaceae and Hyphomicrobiaceae. Additionally, Immundisolibacteraceae and Frankia were abundant in the riparian wetland and alpine meadow soils at Site 6, respectively. However, as shown in Figure 2B, the community composition similarities among riparian wetland sites were higher than those in alpine meadows. The variations in soil diazotrophic community profiles showed significant correlations with multiple environmental factors (Figure 2B). The environmental properties generally showed more significant correlations with soil diazotrophic community in the riparian wetlands than those in the alpine meadows (*P* < 0.05; Figure 2B, Supplementary Table 2 and Supplementary Figure 3). Soil microbial biomass carbon, total organic carbon, moisture, pH, and plant coverage were recognized as key factors shaping the soil diazotrophic community.

### The soil diazotrophic community assembly in the riparian wetlands and their adjacent alpine meadows

**FIGURE 3:**
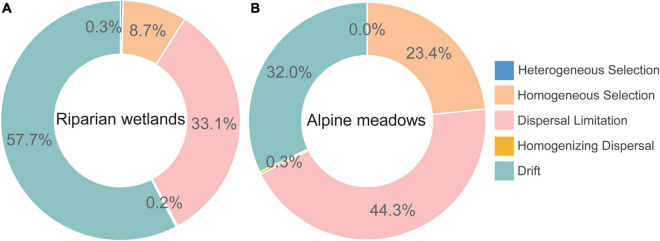
The assembly mechanisms of soil diazotrophic communities in the **(A)** riparian wetlands and **(B)** alpine meadows.

The iCAMP analysis showed that the role of dispersal limitation in driving the assembly of diazotroph communities in alpine meadows (44.3%) was much greater than that in the riparian wetlands (33.1%; Figure 3). Homogeneous selection also contributed much more to the alpine meadow diazotrophic community assembly (23.4%) than that in the riparian wetlands (8.7%). However, drift dominantly controlled the soil diazotrophic community assembly in the riparian wetland soils (57.7%), and its contribution was much higher than that in the alpine meadows (32.0%). Additionally, the contributions of heterogeneous selection and homogenizing dispersal to the soil diazotrophic community assembly were almost negligible in both riparian wetlands and their adjacent alpine meadows.

### The soil diazotrophic functional profiles and their relationships with environmental factors

**FIGURE 4:**
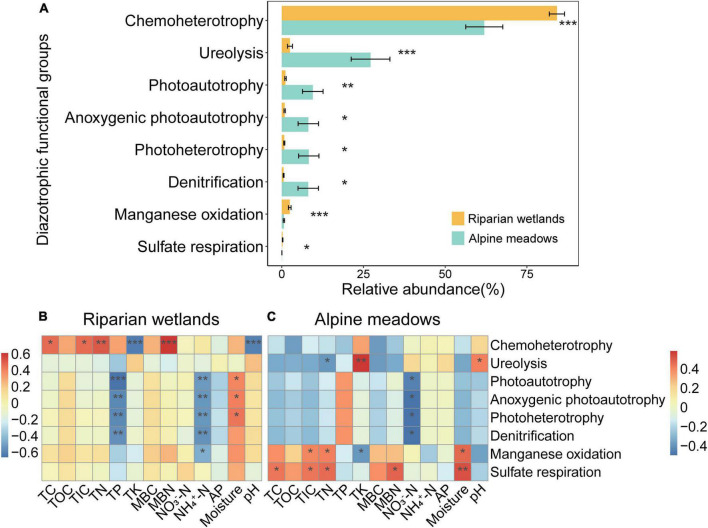
The functional profiles of soil diazotrophic communities. **(A)** The relative abundance of diazotrophic functional groups in the riparian wetland and alpine meadow soils. Only the functional groups showed significant differences are shown. All the data were presented as mean ± SE. **(B)** The relationships between the relative abundance of soil diazotrophic functional groups and soil properties in the riparian wetlands. **(C)** The relationships between the relative abundance of soil diazotrophic functional groups and soil properties in the alpine meadows. TC, soil total carbon content; TOC, soil total organic carbon content; TIC, soil total inorganic carbon content; TN, soil total nitrogen content; TP, soil total phosphorus content; TK, soil total potassium content; MBC, soil microbial biomass carbon content; MBN, soil microbial biomass nitrogen content; NO_3_^–^-N, soil nitrate nitrogen content; NH_4_^+^-N, soil ammonium nitrogen content; AP, soil available phosphorus content, and moisture: soil moisture content. Asterisks indicate the significance of the ANOVA results (*P < 0.05; **P < 0.01; and ***P < 0.001).

The FAPROTAX predictions showed that most of the diazotrophs were multifunctional, and the relative abundance of 8 soil diazotrophic functional groups showed significant differences between the riparian wetlands and their adjacent alpine meadows (Figure 4A; *P* < 0.05). As shown in Figure 4A, the diazotrophs related to chemoheterotrophy, manganese oxidation, and sulfate respiration significantly enriched in the riparian wetland soils. However, the proportions of diazotrophs related to ureolysis, photoautotrophy, anoxygenic photoautotrophy, photoheterotrophy, and denitrification were significantly higher in the alpine meadow soils.

The proportions of soil diazotrophic functional groups were significantly correlated with multiple environmental factors (Figures 4B,C). In the riparian wetlands (Figure 4B), the relative abundance of chemoheterotrophic diazotrophs showed positive correlations with soil total carbon, total inorganic carbon, total nitrogen, and microbial biomass nitrogen contents, while it was negatively correlated with soil total potassium content and pH (*P* < 0.05). The contents of soil total phosphorus and ammonium nitrogen were negatively correlated with the relative abundance of diazotrophs related to photoautotrophy, anoxygenic photoautotrophy, photoheterotrophy, and denitrification (*P* < 0.01). In contrast, the relative abundance of photoautotrophic, anoxygenic photoautotrophic, and photoheterotrophic diazotrophs was positively correlated with soil moisture content (*P* < 0.05). In the alpine meadow soils (Figure 4C), the ureolysis diazotrophic proportions were positively correlated with soil total potassium content and pH, and negatively correlated with soil total nitrogen contents (*P* < 0.05). The content of soil nitrate nitrogen was negatively correlated with the relative abundance of diazotrophs related to photoautotrophy, anoxygenic photoautotrophy, photoheterotrophy, and denitrification (*P* < 0.05). The relative abundance of manganese-oxidation diazotrophs showed positive correlations with the contents of soil total inorganic carbon, total nitrogen, and moisture and negative correlation with the content of total potassium (*P* < 0.05). Additionally, there were positive correlations between sulfate-respiration diazotroph proportions and the contents of soil total carbon, total inorganic carbon, total nitrogen, microbial biomass nitrogen, and moisture (*P* < 0.05).

## Discussion

We found that the abundance and richness of soil diazotrophs in the glacial riparian wetlands were significantly higher than those in their adjacent alpine meadows (Figure 1). This is also supported by a study which has identified wetlands as the hotspots of soil diazotrophs (Che et al., 2018a). The reasons for this phenomenon may be as follows. First, the glacial river promotes microbial dispersal and directly increases the richness and abundance of diazotrophs in the riparian wetlands (Luo et al., 2020). Particularly, the microbial community coalescence in the riverine ecosystem can further increase diazotrophic diversity (Rillig et al., 2015; Mansour et al., 2018; Rocca et al., 2020). Second, as observed in multiple studies, diazotrophs are copiotrophic and usually more abundant in the environments with high organic carbon content (Che et al., 2018b,^2019^; Hu et al., 2020; Li et al., 2021b). Thus, the higher abundance of soil diazotrophs in the glacial riparian wetlands could be elicited by their significantly higher plant productivity and soil organic carbon contents (Supplementary Table 1 and Supplementary Figure 2). Moreover, according to the “more-individuals hypothesis,” the higher availability of energy could also cause the increase in soil diazotrophic diversity (Storch et al., 2018). Finally, compared with the alpine meadows, the habitat heterogeneity (i.e., patchiness caused by flooding degree) of the riparian wetlands is usually much higher due to highly diverse redox potentials and many other environmental properties. This could also contribute to its significantly higher soil diazotrophic richness (Supplementary Table 1 and Supplementary Figure 2). As for the richness difference among the sites, this can be explained by the interaction effects of environmental selection and dispersal limitation on soil diazotrophic richness (Figure 1B). Specifically, the upstream soils were enriched in nutrients such as organic carbon, which can support diazotrophic community with higher diversity. However, as observed in many studies, the downstream riparian areas are less affected by dispersal limitation, which can directly increase the diazotrophic diversity (Zhang et al., 2022). Consequently, the lowest diazotrophic richness was observed in the midstream region (Figure 1B). Additionally, the different soil types can be another reason for the higher soil diazotrophic richness at the first two sites than that in the other sites. These findings indicate that the expansion and retreat of riparian wetlands probably will increase and decrease the diazotrophic abundance and richness, respectively. This may further affect nitrogen inputs from biological nitrogen fixation.

The community composition of diazotrophs in the glacial riparian wetlands and alpine meadows also significantly differed (Figure 2). This discrepancy can be also mainly elicited by the large differences in plant and soil properties between the glacial riparian wetlands and alpine meadows (Supplementary Table 1 and Supplementary Figure 2). Indeed, most of these environmental factors showed significant correlations with soil diazotrophic community profiles (Figure 2B, Supplementary Table 2 and Supplementary Figure 3). Moreover, the critical roles of plant community profiles, productivity, soil pH, and the contents of soil moisture and nutrients have been recognized as key factors shaping soil diazotrophic communities in multiple investigations (Wang et al., 2017; Che et al., 2018a; Cao et al., 2022). Additionally, as one of the main components of biological crusts, Scytonemataceae was more abundant in the alpine meadows which was consistent with our previous study (Che et al., 2018a). Collectively, the differences in soil diazotrophic community profiles between the glacial riparian wetlands and alpine meadows could be the consequences of the interaction between the environmental factors and habitat preference.

In this study, the assembly of diazotrophic communities in the riparian wetland soils was much less affected by dispersal limitation compared with that in their adjacent alpine meadows (Figure 3). The dispersal of soil microbes in the environment mainly depends on external forces (Gammack et al., 2021). Hence, the differences in the assembly mechanisms of soil diazotrophic communities in the riparian wetlands and their adjacent alpine meadows can be mainly attributed to the environmental connectivity and fluidity (Zhang et al., 2022). Specifically, compared with the alpine meadows, the riparian wetlands were close to the river, and the higher environmental connectivity and fluidity prompted microbial dispersal (Wang et al., 2020a). This result is in accordance with our recent study along the Nu River (Zhang et al., 2022), and also explains the higher community composition similarities among the riparian wetland sites than those in the alpine meadows (Figure 2B). Additionally, the higher contribution of homogeneous selection to the alpine meadow diazotrophic community assembly may be induced by the lower environmental heterogeneity of alpine meadows compared with the riparian wetlands (Figure 3, Supplementary Table 1 and Supplementary Figure 2). We also found that drift is the more predominant process controlling the assembly of soil diazotroph communities in the riparian wetlands than that in the alpine meadows (Figure 3). In the iCAMP analysis, drift is defined as a collection of genetic drift, diversification, weak selection, and/or weak dispersal (Ning et al., 2020). Its dominant role in diazotrophic community assembly can be partially explained by the lower disturbance caused by human activities (e.g., grazing) and historical accumulation (i.e., original niche occupation) in the riparian wetlands compared with alpine meadows (Shi et al., 2010). However, the large contribution of stochastic process also highlights the challenges in modeling and predicting soil diazotrophic community profiles in the alpine ecosystems.

Another finding in this study was that the soil diazotrophic multiple-functionality also significantly differed between the glacial riparian wetlands and alpine meadows (Figure 4A). For example, compared with the alpine meadows, the chemoheterotrophic diazotrophs were more abundant in riparian wetland soils. This could be mainly caused by the enrichments of organic carbon and other nutrients such as microbial biomass (Supplementary Table 1 and Supplementary Figure 2; Ping et al., 2021). Moreover, compared with riparian wetlands, the alpine meadows are usually much more heavily grazed by livestock. This can lead to the increase in feces deposition, the removal of plant biomass, and light availability, thereby favoring the diazotrophs related to ureolysis and photoautrophy (Figure 4A; Luo et al., 2009). These findings suggest that the interconversion between glacial riparian wetlands and alpine meadows can also substantially modify the coupling relationships between nitrogen fixation and other ecological functions. However, these conclusions should be further verified by the future studies with metagenomic analysis which can directly analyze diazotrophic functions.

Manifold environmental factors such as soil moisture, pH, total nitrogen, and total potassium were significantly correlated with the relative abundance of soil diazotrophic functional taxa in the riparian wetlands and alpine meadows (Figures 4B,C). Indeed, the critical roles of these factors in shaping soil diazotrophic community have been widely recognized (Tang et al., 2017; Che et al., 2018a; Xiao et al., 2020; Zhang et al., 2021a). In the riparian wetlands, chemoheterotrophic microbes are highly dependent on the availability of nutrients, which can be the main reason for the positive correlations between the proportions of chemoheterotrophic diazotrophs and the contents of soil carbon and nitrogen (Figure 4B). The significantly negative correlations between photoautotrophic diazotroph proportions and the contents of soil total phosphorus and ammonium nitrogen may be ascribed to the higher plant cover and lower light availability under better nutrient conditions (Scognamiglio et al., 2021). As for the alpine meadows, denitrification is the process of reducing nitrate, and thus the relative abundance of denitrification diazotrophs and soil nitrate nitrogen content were negatively correlated (Figure 4C; Kuypers et al., 2018). Manganese oxidation has been proven to fuel the growth of chemolithoautotrophic microorganisms and carbon fixation (Yu and Leadbetter, 2020). It has also been proposed that manganese oxidation processes in bacteria may be part of the pathway for microbial uptake of metabolic nutrients and energy-producing metabolism, and facilitate microbial adaptation to external stresses (Duan et al., 2020). These may be the reasons for the significantly positive correlations between the relative abundance of manganese-oxidation diazotrophs and the contents of soil total inorganic carbon and total nitrogen. Additionally, sulfate respiration was an important anaerobic carbon oxidation pathway, which explained the positive correlations between the proportions of diazotrophs related to sulfate respiration and the contents of soil moisture, total carbon, and total inorganic carbon (Rysgaard et al., 2001). However, we also noticed that the environmental factors showed markedly different relationships with the relative abundance of riparian wetland and alpine meadow soil diazotrophic functional taxa (Figures 4B,C). Overall, the contents of total phosphorus and ammonium nitrogen were the main predictors of soil diazotrophic functional profiles in the riparian wetlands, while the alpine meadow soil diazotrophic functional profiles can be mainly predicted by soil nitrate nitrogen content. These inconsistent relationships can be caused in several ways. First, they can be ascribed to the significantly different diazotrophic community profiles between the riparian wetlands and alpine meadows (Figure 2). Second, the different spatial trends of the riparian wetland and alpine meadow environmental variables among the sites may also play certain roles (Supplementary Table 1 and Supplementary Figure 2). Finally, it has been proven that different ecosystems have different limiting factors for the same ecological process, which can be another potential reason for the aforementioned inconsistent relationships (Li et al., 2021c). These results suggest that the coupling between biological nitrogen fixation and other ecosystem functions can be modified by multiple environmental factors, and the regulating mechanisms are probably distinct between riparian wetlands and alpine meadows.

## Conclusion

This study showed that the soil diazotrophic abundance and richness in glacial riparian wetlands were significantly higher than those in their adjacent alpine meadows. The profiles and assembly mechanisms of soil diazotrophic community between the glacial riparian wetlands and their adjacent alpine meadows also significantly differed. Specifically, the diazotrophs related to ureolysis, photoautotrophy, and denitrification were more abundant in the alpine meadows, while chemoheterotrophic and sulfate-respiration diazotrophs were significantly enriched in the riparian wetlands. In addition, we also found that the assembly of diazotrophic communities was mainly controlled by drift and dispersal limitation. Compared to the glacial riparian wetlands, dispersal limitation and homogeneous selection contributed more to the soil diazotrophic community assembly in the alpine meadow soils. Collectively, these findings suggest that the expansion and retreat of the riparian wetlands under the scenario of global warming can substantially affect ecosystem nitrogen input mechanisms through altering soil diazotrophic community.

## Data availability statement

The datasets presented in this study can be found in online repositories. The names of the repository/repositories and accession number(s) can be found in the article/Supplementary material.

## Author contributions

DC: data curation, formal analysis, and writing—original draft preparation. RC: conceptualization, methodology, and writing—reviewing and editing. HH: formal analysis and writing—original draft preparation. SZ: methodology. StZ and ZP: investigation. DL: writing—reviewing and editing. JH, KX, JD, XC, and YW: funding acquisition and project administration. All authors contributed to the article and approved the submitted version.
