# Supplementary material for: Soil diazotrophic abundance, diversity, and community assembly mechanisms significantly differ between glacier riparian wetlands and their adjacent alpine meadows
Source: Front Microbiol. 2022 Dec 8;13:1063027. doi: 10.3389/fmicb.2022.1063027 (PMC9772447; doi:10.3389/fmicb.2022.1063027)
Supplement: Supplementary file 1 [file Data_Sheet_1.PDF]

Supplementary Table 1. Soil and plant properties of the riparian wetlands and their adjacent alpine meadows

Properties	TC (g/kg)	TOC (g/kg)	TIC (g/kg)	TN (g/kg)	TP (g/kg)	TK (g/kg)	MBC (mg/kg)	MBN (mg/kg)
R	48.420± 4.585a	11.639± 0.592a	36.781± 4.230a	3.831± 0.355a	0.744± 0.022a	20.787± 0.548a	103.400± 5.029a	116.209± 8.575a
A	35.689± 5.048a	8.743± 0.740b	26.946± 4.406a	2.782± 0.392a	0.757± 0.032a	22.316± 0.543a	78.856± 6.429b	80.632± 8.724b

Properties	NO_3^--N (mg/kg)	NH_4^+-N (mg/kg)	AP (mg/kg)	Moisture	pH	PC (%)	PR	PB (g/m^2)
R	6.880± 0.174a	45.836± 2.119a	5.996± 0.283a	0.478± 0.040a	6.533± 0.159a	71.875± 3.442a	10.222± 0.417a	112.348± 10.918a
A	6.080± 0.202b	45.763± 2.571a	6.332± 0.160a	0.233± 0.044b	6.643± 0.197a	44.706± 7.038b	9.778± 1.015a	86.458± 13.653a

Notes: All the properties are presented as means ± standard error. The groups without sharing the same letters indicate significant differences (Duncan's test, $P < 0.05$). R: riparian wetlands, A: alpine meadows. TC: soil total carbon content, TOC: soil total organic carbon content, TIC: soil total inorganic carbon content, TN: soil total nitrogen content, TP: soil total phosphorus content, TK: soil total potassium content, MBC: soil microbial biomass carbon content, MBN: soil microbial biomass nitrogen content, NO_3^--N: soil nitrate nitrogen content, NH_4^+-N: soil ammonium nitrogen content, AP: soil available phosphorus content, and moisture: soil moisture content. PC: plant coverage, PR: plant richness, and PB: plant biomass.

Supplementary Table 2. The results of the envfit analysis of diazotrophic community structure in the riparian wetland and the alpine meadow soils

Envfit	Result	TC	TOC	TIC	TN	TP	TK	MBC	MBN	NO_3^--N	NH_4^+-N	AP	Moisture	pH	PC	PR	PB
Figure 2b	r^2	0.2065	0.4532	0.1602	0.2217	0.0162	0.1886	0.5210	0.2495	0.0755	0.0003	0.0682	0.4240	0.3270			
	P	0.001	0.001	0.008	0.001	0.649	0.005	0.001	0.001	0.145	0.994	0.166	0.001	0.001			
Figure S3	r^2	0.2394	0.6108	0.1915	0.2431	0.0399	0.3631	0.5958	0.3339	0.1234	0.0747	0.0940	0.4399	0.4287	0.3786	0.1488	0.0518
	P	0.011	0.001	0.026	0.013	0.515	0.001	0.001	0.003	0.118	0.262	0.191	0.001	0.001	0.001	0.066	0.406

Notes: TC: soil total carbon content, TOC: soil total organic carbon content, TIC: soil total inorganic carbon content, TN: soil total nitrogen content, TP: soil total phosphorus content, TK: soil total potassium content, MBC: soil microbial biomass carbon content, MBN: soil microbial biomass nitrogen content, NO_3^--N: soil nitrate nitrogen content, NH_4^+-N: soil ammonium nitrogen content, AP: soil available phosphorus content, and moisture: soil moisture content. PC: plant coverage, PR: plant richness, and PB: plant biomass.

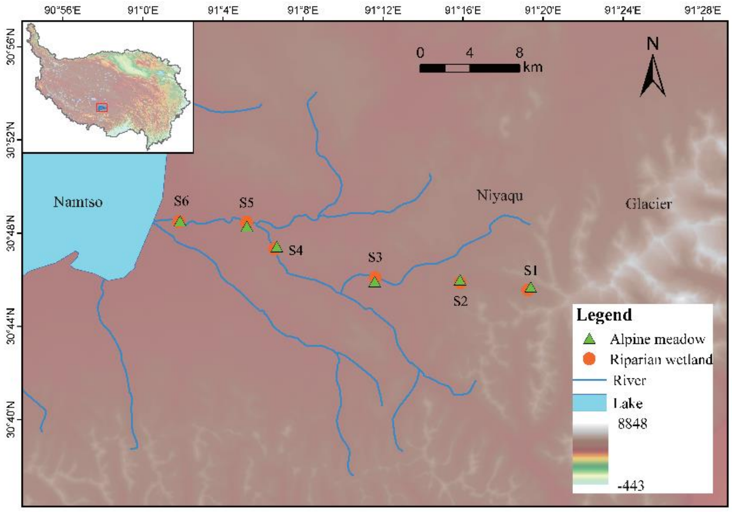

Supplementary Figure 1. The distribution of sampling sites.

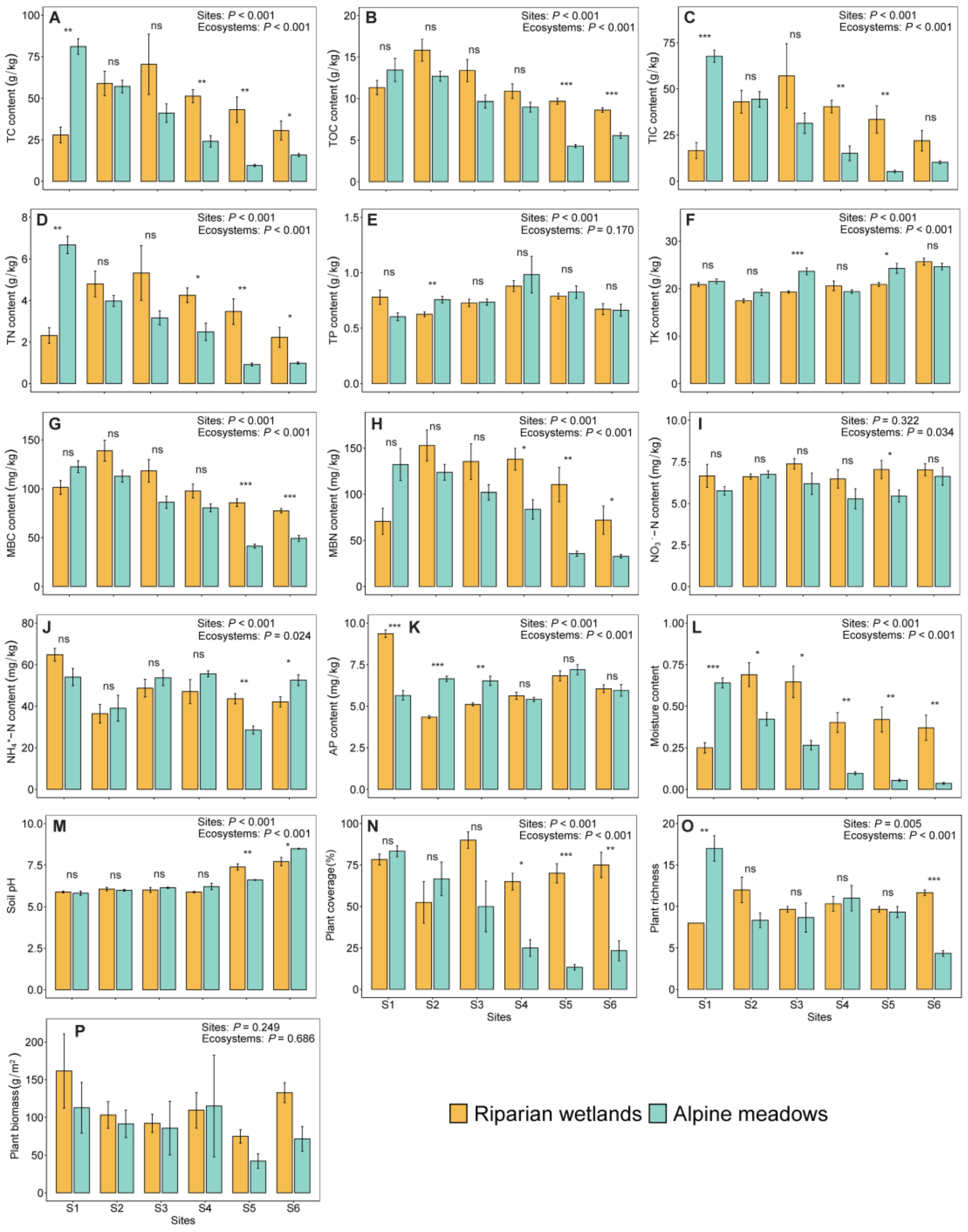

Supplementary Figure 2. The differences in soil and plant properties between the riparian wetlands and their adjacent alpine meadows. All the data were presented as mean ± SE. Asterisks indicate the significance of the ANOVA results (*: $P < 0.05$; **: $P < 0.01$; ***: $P < 0.001$; ns: no significance). TC: soil total carbon content, TOC: soil total organic carbon content, TIC: soil total inorganic carbon content, TN: soil total nitrogen content, TP: soil total phosphorus content, TK: soil total potassium content, MBC: soil microbial biomass carbon content, MBN: soil microbial biomass nitrogen content, NO_3^--N: soil nitrate nitrogen content, NH_4^+-N: soil ammonium nitrogen content, AP: soil available phosphorus content, and moisture: soil moisture content.

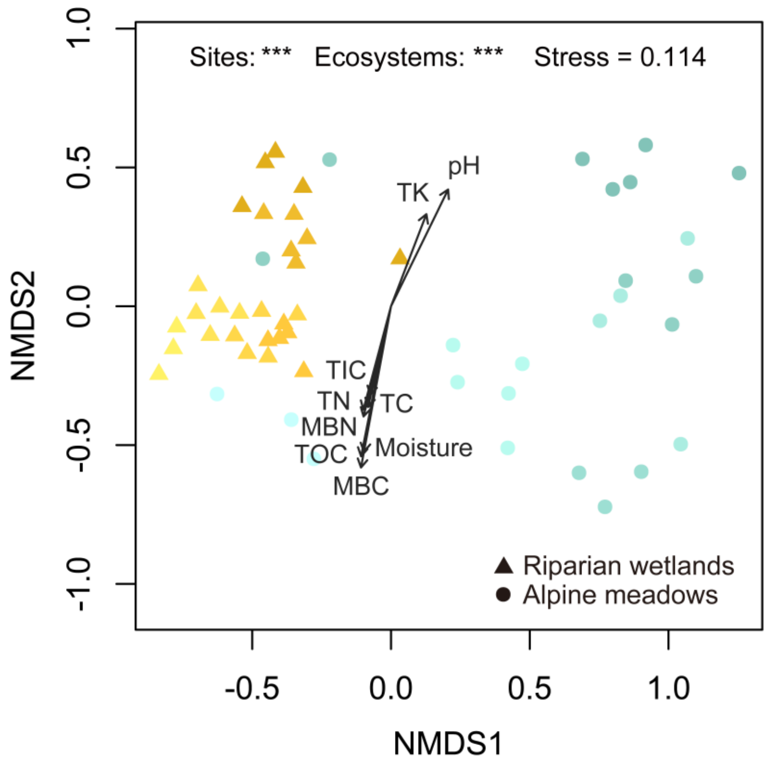

Supplementary Figure 3. The NMDS ordination combined with envfit analysis of diazotrophic community structure in the riparian wetland and the alpine meadow soils. TC: soil total carbon content, TOC: soil total organic carbon content, TIC: soil total inorganic carbon content, TN: soil total nitrogen content, TK: soil total potassium content, MBC: soil microbial biomass carbon content, MBN: soil microbial biomass nitrogen content, and moisture: soil moisture content.

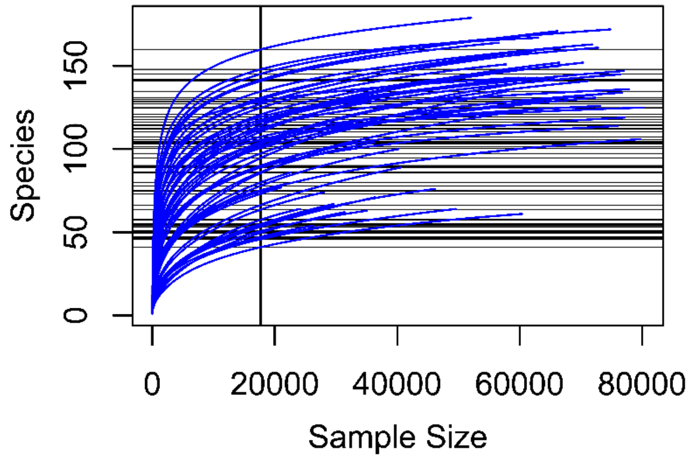

Supplementary Figure 4. Rarefaction curve of diazotrophic OTUs detected in the riparian wetland and the alpine meadow soils.
